# Single-session robotic bronchoscopy diagnoses histologically discordant synchronous lung lesions

**DOI:** 10.1093/omcr/omag204

**Published:** 2026-11-02

**Authors:** Venkatkiran Kanchustambham, Swetha Saladi, Alexey Hodkoff

**Affiliations:** Department of Internal Medicine, University of North Dakota School of Medicine and Health Sciences, 1301 N Columbia Rd, Grand Forks, ND 58203, United States; Division of Pulmonary and Critical Care Medicine, Sanford Health, 5225 23rd Ave S, Fargo, ND 58104, United States; Department of Internal Medicine, University of North Dakota School of Medicine and Health Sciences, 1301 N Columbia Rd, Grand Forks, ND 58203, United States; Department of Internal Medicine, Sanford Health, 5225 23rd Ave S, Fargo, ND 58104, United States; Department of Pathology, Sanford Health, 5225 23rd Ave S, Fargo, ND 58104, United States

**Keywords:** discordant histology, histologic transformation, multiple primary lung cancer, non-small cell lung cancer, robotic bronchoscopy, small cell lung cancer

## Abstract

Histologically discordant synchronous pulmonary lesions in a previously treated patient may represent independent primaries, intrapulmonary metastasis, or histologic transformation. A 74-year-old woman with previously treated right upper lobe adenosquamous carcinoma developed two new, spatially separate, solid fluorodeoxyglucose-avid lesions. Both were sampled during one anaesthetic using shape-sensing robotic-assisted bronchoscopy with radial-probe endobronchial ultrasound confirmation. The peripheral right upper lobe lesion was small cell carcinoma; the left upper lobe lesion was non-small cell carcinoma, not otherwise specified, with patchy TTF-1 and p40 expression compatible with adenosquamous differentiation. Comparative clonality testing was not performed, so their relationship remained unproven. Both lesions received stereotactic ablative radiotherapy (50 Gy in five fractions); the small cell carcinoma was also treated with carboplatin and etoposide, with consolidation durvalumab planned. Bilateral sampling revealed clinically consequential histologic heterogeneity and enabled histology-directed therapy without assuming a common origin.

## Introduction

Patients with multiple pulmonary lesions may harbour tumours of different biology and treatment requirements. Concurrent small cell lung carcinoma (SCLC) and non-small cell lung carcinoma (NSCLC) are uncommon [1, 2]. Classification is particularly difficult after prior lung-cancer therapy because a new SCLC lesion may represent an independent primary, intrapulmonary metastasis, or neuroendocrine transformation of the treated tumour [3, 4]. Histology, imaging distribution, treatment history, and molecular comparison should therefore be integrated rather than relying on anatomic distribution alone [5]. Shape-sensing robotic-assisted bronchoscopy (ssRAB) can reach separate peripheral targets during one anaesthetic, and single-session diagnosis of discordant synchronous histology has been reported [6]. We describe bilateral histologically discordant synchronous lesions, SCLC and NSCLC, diagnosed in this manner in a previously treated patient.

## Case report

A 74-year-old woman was referred for evaluation of new bilateral pulmonary lesions. She had a smoking history exceeding 50 pack-years, chronic obstructive pulmonary disease, and a paternal family history of lung cancer. Her oncologic history included right upper lobe adenosquamous carcinoma treated by wedge resection in 2019; recurrence with station 4R nodal involvement treated with definitive chemoradiotherapy in 2021; adjuvant immune-checkpoint inhibitor therapy in 2022; and ablative radiotherapy in 2023 for a presumed local recurrence adjacent to the prior resection bed, without tissue confirmation. Functional status remained preserved.

Surveillance computed tomography in March 2026 showed post-treatment change in the medial right upper lobe but no discrete peripheral right upper lobe nodule (Fig. 1A). Fluorodeoxyglucose positron-emission tomography/computed tomography in July 2026 demonstrated two new, intensely avid, spatially separate solid lesions: a peripheral right upper lobe nodule measuring 1.5 cm and a left upper lobe lesion measuring 1.3 cm (maximum standardised uptake values 16.6 and 21.6, respectively), without overt mediastinal or hilar adenopathy (Fig. 1B-E). The dimensions represent total radiographic diameters. The peripheral right lesion arose in previously uninvolved lung remote from the treated medial right upper lobe field. Contrast-enhanced magnetic resonance imaging of the brain showed no intracranial metastases.

**Figure 1:**
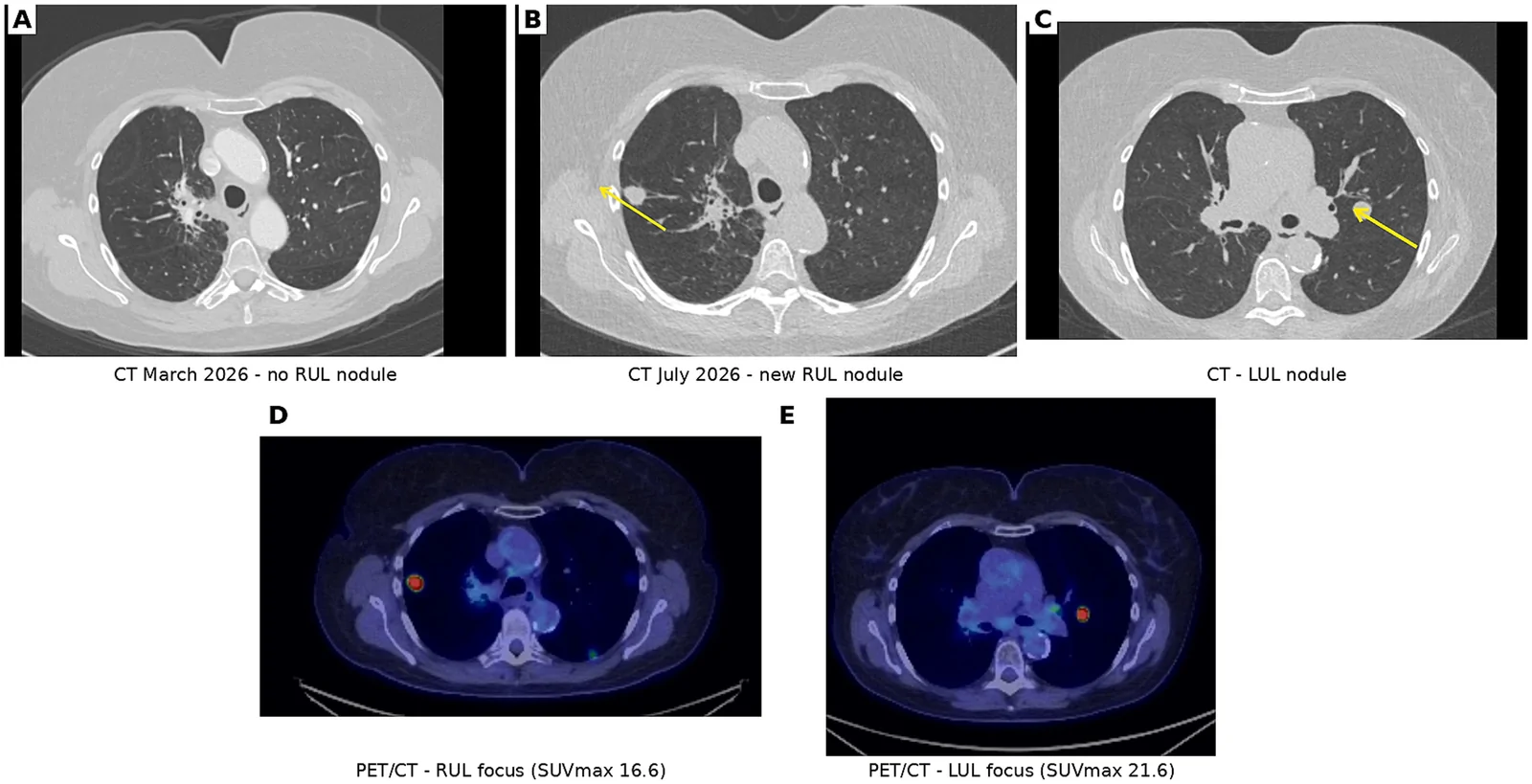
Serial CT and PET/CT. (A) March 2026: no peripheral right upper lobe nodule; medial change reflects the treated 2019 wedge/2023 ablative-radiotherapy bed. (B) July 2026: Interval development of a discrete peripheral right upper lobe nodule (arrow; small cell carcinoma). (C) Left upper lobe nodule (arrow; non-small cell carcinoma). (D, E) fused FDG-PET/CT: Two hypermetabolic, spatially separate foci (SUVmax 16.6 and 21.6) without intervening nodal uptake.

Both lesions were sampled during one procedure under general anaesthesia using ssRAB (Ion endoluminal system; Intuitive Surgical, Sunnyvale, California, USA) (Fig. 2A and B). Radial-probe endobronchial ultrasound demonstrated concentric within-lesion signals at both targets before sampling (Fig. 2C and D). A systematic linear EBUS survey examined stations 11 L, 4 L, 7, 4R, and 11R. Stations 7, 4R, and 11R were sampled on the basis of nodal size and sonographic appearance; stations 4 L and 11 L measured less than 5 mm and were not sampled. All sampled stations were negative for malignancy; station 4R showed reactive changes with a loose granuloma. No procedural complications occurred.

**Figure 2:**
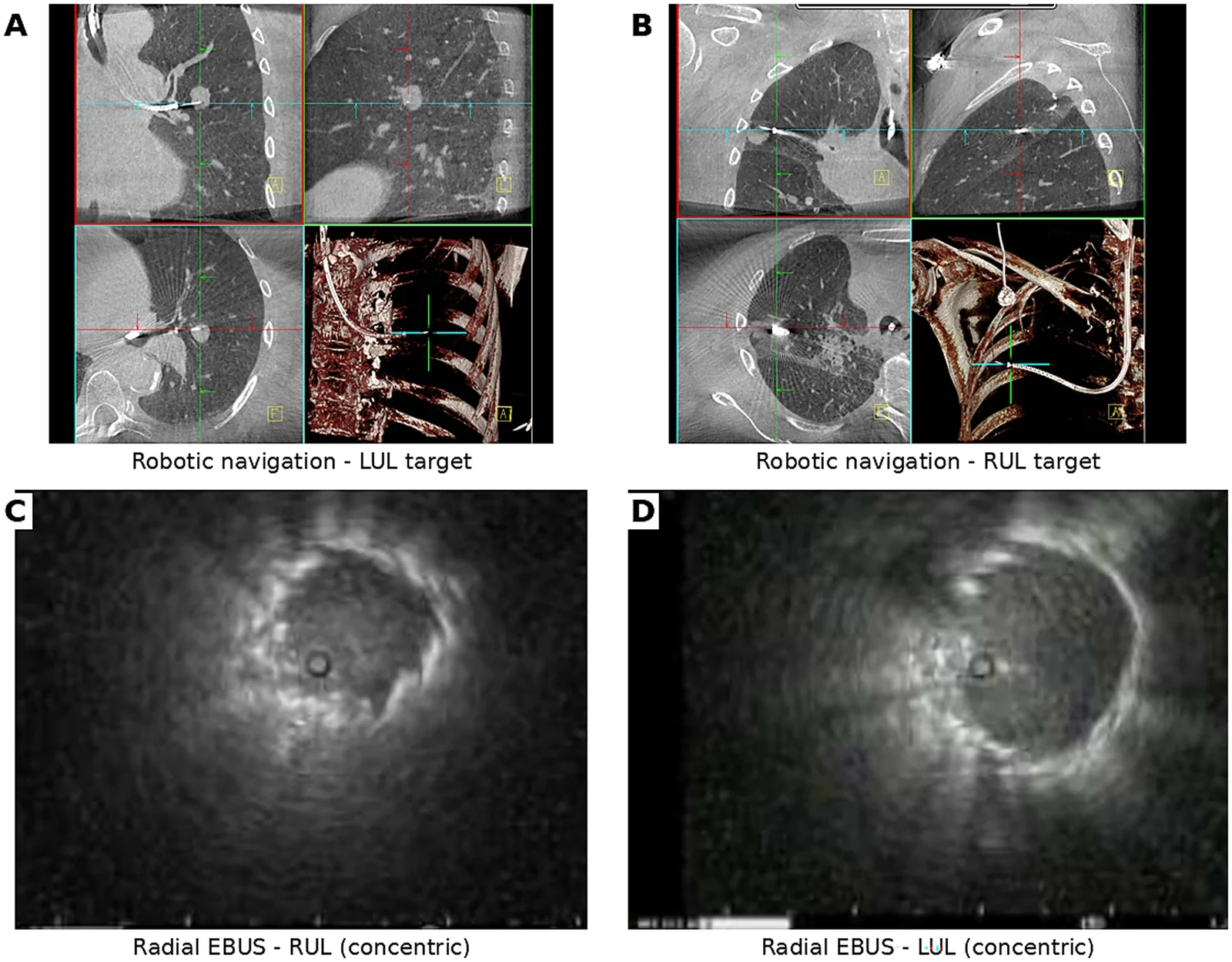
Single-session robotic navigation and radial EBUS. (a, B) ssRAB navigation captures demonstrating tool-in-lesion position at the left (A) and right (B) upper lobe targets. (C, D) radial-probe EBUS shows concentric within-lesion signals at the right (C) and left (D) upper lobe targets. EBUS-guided sampling of stations 7, 4R, and 11R was negative for malignancy.

The right upper lobe lesion was diagnosed as SCLC. The institutional report did not separately describe classic small-cell morphology. Neoplastic cells expressed pancytokeratin, CAM5.2, CD56, and synaptophysin; TTF-1, p40, CD99, and chromogranin were negative; INI-1 (SMARCB1) expression was retained; and the Ki-67 labelling index exceeded 80%. INSM1 was not performed (Fig. 3A-D). The left upper lobe lesion was NSCLC, not otherwise specified. Glandular and squamous morphology were not separately identified on H&E, and the classification rested primarily on patchy TTF-1 and p40 expression, with absent CD56 and chromogranin, compatible with adenosquamous differentiation and the phenotype of the prior carcinoma (Fig. 3E-H).

**Figure 3:**
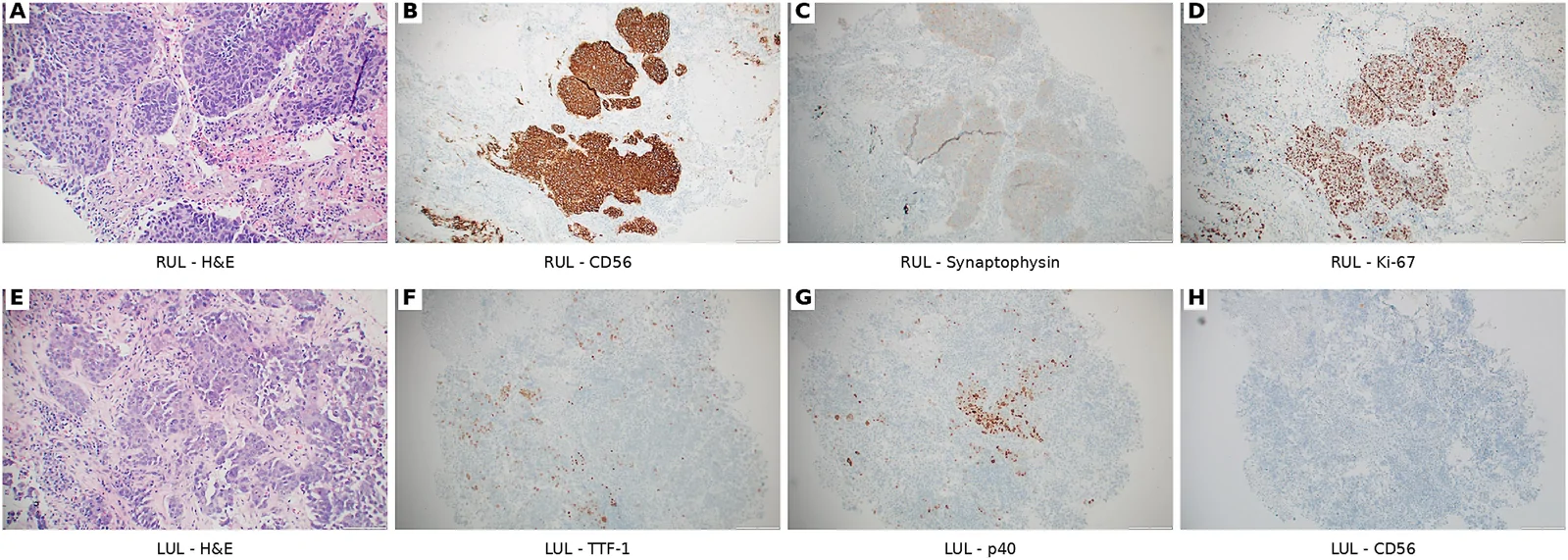
Discordant histology. Right upper lobe lesion (A-D): (A) H&E; (B) CD56; (C) synaptophysin; (D) Ki-67, supporting small cell carcinoma. Left upper lobe lesion (E-H): (E) H&E; (F) TTF-1; (G) p40; (H) CD56, supporting non-small cell carcinoma with patchy glandular and squamous marker expression. Scale bars as annotated.

The multidisciplinary tumour board favoured synchronous histologically discordant pulmonary malignancies while recognising that an independent SCLC primary, transformation, and metastatic relationships could not be distinguished definitively. Comparative clonality testing was not pursued because residual tissue after diagnostic immunohistochemistry was limited and comparison would also have required archived tumour material. Using the ninth edition of the TNM classification, the right upper lobe SCLC and left upper lobe NSCLC were each clinically staged cT1bN0M0 (stage IA2); the SCLC also met the Veterans Administration definition of limited-stage disease. Surgical resection was not planned. Both lesions received stereotactic ablative radiotherapy, 50 Gy in five 10-Gy fractions, from 18 to 28 August 2026. The SCLC was additionally treated with carboplatin (AUC 5) and etoposide (100 mg/m2), initiated with radiotherapy; by 22 September 2026, two of four planned cycles had been administered. Consolidation durvalumab for up to 24 months was planned after chemoradiotherapy if there was no progression [7]. The first chemotherapy cycle was tolerated well, and no unexpected radiotherapy toxicity occurred. Chest CT and brain MRI were scheduled for 19 November 2026; response imaging was not yet available.

## Discussion

This case highlights the risk of assigning a single diagnosis to multifocal pulmonary disease in a patient with previously treated lung cancer. Sampling only the left lesion could have led to treatment as recurrent NSCLC, whereas sampling only the right lesion could have obscured the simultaneously active NSCLC component. Obtaining tissue from both radiographically distinct targets demonstrated clinically consequential histologic heterogeneity. Single-session robotic diagnosis of discordant synchronous histology has been described previously [6, 8], but not, to our knowledge, for an SCLC and NSCLC pair in a previously treated patient.

Small cell transformation remained an important alternative because the patient had received chemoradiotherapy and immune-checkpoint inhibition. Transformation is best characterised in oncogene-driven adenocarcinoma after targeted therapy but has also been reported after immunotherapy and in tumours without an actionable driver [3, 4, 9]. Several features favoured an independent SCLC primary: the lesion was absent four months earlier and formed a discrete hypermetabolic focus in previously uninvolved lung. Location alone cannot exclude transformation, however, and these features support but do not prove an independent origin.

Traditional Martini-Melamed criteria regard major histologic discordance as evidence of separate primaries [10]. Contemporary IASLC guidance is more cautious, treating discordant histology as suggestive rather than definitive and integrating detailed histology, radiographic behaviour, nodal and distant spread, and molecular profiles [5]. The left upper lobe NSCLC could represent a new primary or contralateral recurrence of the prior adenosquamous carcinoma. Likewise, definitive separation of a new SCLC primary from transformation would require molecular comparison of both current lesions with archived tumour tissue. Substantially divergent profiles would support independent tumours, whereas shared alterations would favour a clonal relationship. Although the biopsy provided adequate cells for the diagnostic immunohistochemical panel, residual tissue for additional comparative profiling was limited. The absence of clonality analysis therefore remains the principal limitation of this report.

The procedural lesson is more generalisable than the final classification. ssRAB uses a pre-procedure CT-derived three-dimensional airway plan and fibre-optic shape sensing to track a steerable catheter during navigation. Compared with conventional flexible bronchoscopy, it offers greater distal reach and catheter stability for peripheral targets and permits sequential sampling of multiple lesions during one anaesthetic [8, 11]. Radial-ultrasound confirmation at each target and documented independent navigation also reduce concern that discordant results reflect sampling error or specimen cross-contamination. Limitations include the need for specialised equipment, operator training, and usually general anaesthesia; CT-to-body divergence may impair localisation, and transbronchial sampling retains risks of bleeding and pneumothorax [11]. When multifocal lesions differ in location, growth pattern, or metabolic behaviour, each clinically relevant lesion should be considered for tissue diagnosis rather than presumed to share one histology.

In conclusion, single-session bilateral ssRAB identified concurrent SCLC and NSCLC and enabled histology-directed treatment in a previously treated patient. The clinical pattern suggested synchronous discordant malignancies, but the lesions cannot be classified definitively as independent primaries without comparative molecular analysis.

## Data Availability

The data supporting the findings of this report are available from the corresponding author on reasonable request. They are not publicly available because of privacy and ethical restrictions.
